# Graphitization by Metal Particles

**DOI:** 10.1021/acsomega.2c06848

**Published:** 2023-01-12

**Authors:** Stuart J Goldie, Karl S Coleman

**Affiliations:** † Department of Chemistry, Durham University, South Road, DurhamDH1 3LE, U.K.; ‡ Department of Chemistry, School of Physical Sciences, University of Liverpool, Peach Street, LiverpoolL69 7ZE, U.K.

## Abstract

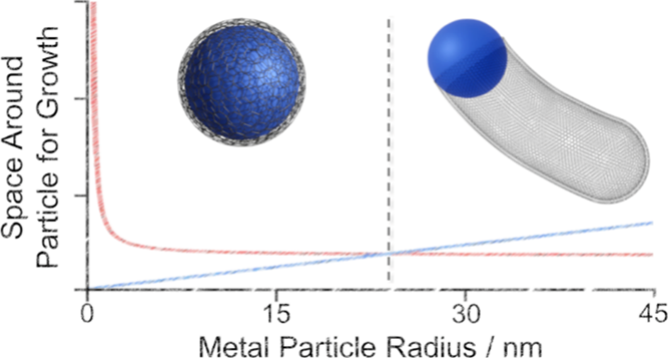

Graphitization of carbon offers a promising route to upcycle waste biomass and plastics into functional carbon nanomaterials for a range of applications including energy storage devices. One challenge to the more widespread utilization of this technology is controlling the carbon nanostructures formed. In this work, we undertake a meta-analysis of graphitization catalyzed by transition metals, examining the available electron microscopy data of carbon nanostructures and finding a correlation between different nanostructures and metal particle size. By considering a thermodynamic description of the graphitization process on transition-metal nanoparticles, we show an energy barrier exists that distinguishes between different growth mechanisms. Particles smaller than ∼25 nm in radius remain trapped within closed carbon structures, while nanoparticles larger than this become mobile and produce nanotubes and ribbons. These predictions agree closely with experimentally observed trends and should provide a framework to better understand and tailor graphitization of waste materials into functional carbon nanostructures.

## Introduction

Graphitization, the process of thermally converting amorphous carbon into graphitic carbon, requires temperatures in excess of 2500 °C and is sensitive to the specific carbon used unless a catalyst is added.^1−4^ Polymers have been extensively investigated owing to their controllable pore structure and tolerance to metal containing additives, for example, resorcinol-formaldehyde emulsions.^5−11^ Biomass-derived carbons have also been used as low cost abundant feedstocks that can be prepared as a wet gel with a metal salt solution or by soaking a metal solution into an insoluble carbon precursor.^12−23^

The conversion of low-cost carbon feedstocks into graphitic materials with a controllable balance of conductivity and hierarchical pore structures has great potential in applications that currently rely on rare and, therefore expensive, minerals. Additionally, in many cases the metal particles add functionality to these materials. Of particular note are energy storage devices such as battery electrodes and supercapacitors, or photo and electro-catalysts.^24−27^ Graphitization has also been used to prepare printable conductive tracks, by selectively printing a metal catalyst^21^ or heating specific areas with laser illumination^28^ in methods that could unlock inexpensive biodegradable electronics.

Unfortunately, controlling the graphitization process remains challenging and the numerous starting materials and process conditions used makes it difficult to draw meaningful conclusions. Were more precise control achieved, it should be possible to design carbon materials with properties matched to applications, balancing low-surface area but highly conductive graphite flakes with high-surface area amorphous carbons.^29,30^ The widespread use of electron microscopy and interest driven by the discovery of graphene provides a volume of literature observations on carbon and metal particle formation that can shape our mechanistic understanding. By collating these results in a meta-analysis of published literature and applying new understanding from the chemical vapor deposition growth of graphitic carbon materials some new insights for graphitization are proposed.

### Metals as Catalysts for Graphitization

Carbon dissolution on heating and subsequent precipitation from cooling metal was the first model proposed to understand catalytic graphitization.^31^ This was furthered in a seminal review where Oya and Marsh highlighted four mechanisms to explain the different carbon materials observed from different catalysts;^4^ G-effect graphitization—observed from large metal particles producing crystalline graphite, *T*_s_-effect—finely divided metal nanoparticles that form turbostratic graphite, A-effect and *T*_n_-effect graphitization—where oxidizing species like CO and some alkali metal vapors react preferentially with defects promoting crystallization of the remaining graphitizable carbon.

Despite the success of these models in explaining many experimental observations, more recent in-situ experiments, have detected graphitic carbon formation during heating rather than upon cooling as expected from the dissolution/precipitation mechanism. High-resolution transmission electron microscopy performed in situ has shown graphenic layers forming around metal particles at 500 °C;^32^ and X-ray diffraction (XRD) shows graphitic carbon formation around 700 °C.^17,33−36^ A surface-mediated rearrangement of carbon into graphitic carbon is clearly more significant than precipitation; especially on smaller metal particles that do not behave as bulk materials, and operating at lower temperatures is more attractive for scalable production.

At these growth temperatures, there also remains some uncertainty regarding the state the metal is in, with discussions of liquid or liquid-like metal particles.^21,37^ This was first reported from transmission electron microscopy (TEM) images of Fe, Ni, and Co flowing through amorphous carbon with no electron diffraction patterns,^38^ far below the eutectic point of the metal-graphite mixtures (e.g., Fe–C = 1153 °C) even when accounting for the nanoparticles size.^10^ However, by taking into account the higher chemical potential of amorphous carbon compared with graphite, Parmon showed such a high carbon solubility into the metal particles is thermodynamically favorable and could result in a meta-stable carbide melt forming at the surface.^39^ A finding supported by other studies on Au and mixed Co/Fe nanoparticles;^40,41^ and recently by in situ XRD of growth around iron particles.^36^

Thus far, we have focused this discussion on elemental metal particles. These are usually prepared from water soluble salts that are easily mixed with carbon templates and reduce to metals when heated with carbon. The relative thermal stability of the salt and carbon material can determine the size and shape of metal particle formed, which in turn exerts considerable influence on the graphitization process.^34,42^ Rapidly reduced salts form small nanoparticles trapped within the carbon, whereas delayed reduction, until the carbon matrix had already decomposed, provides space for the metal to anneal. In the case of Fe particles, the size of the oxide particles initially formed is also responsible for the differentiation between carbide or metal after reduction.^43^

In addition to the thermal stability of the metal precursors and carbon material, the structure before any heating must be acknowledged. A commonly used method to control the initial structure is soft templating because it easily facilitates the addition of metal salts. Emulsion polymerization is a very popular soft templating method because of the precise control achieved, this has been extensively reviewed elsewhere.^44−46^ Of particular note are resorcinol-formaldehyde foams,^47^ used extensively because of the ease of production by using acidic metal salts to initiate the polymerization. These hardened foams are then readily graphitized with the metal salts transforming into metal particles.

Another very attractive option is the use of waste biomass. Templating of a hierarchical porous structure is achieved by forming a wet gel that can be dried into a hard foam, or impregnation of insoluble materials with a metal salt solution. Examples of materials graphitized this way include: sawdust,^12^ kraft lignin,^14,15^ saccharides,^16,17^ peanut shells,^18^ whey protein,^19^ poplar wood,^20^ cellulose paper,^21^ polypeptides,^22,23^ and non-biomass heavy hydrocarbon waste.^28,48^

In addition to the economic benefit of using waste materials, many biomass-derived carbon sources readily carbonize into highly porous materials, even in the absence of metal catalysts, and contain a wide range of functional groups that influence the distribution of metal ions when they are used.^17,22,43^ Different materials are known to produce various hierarchical pore structures allowing further tailoring of the final material.^49,50^

Graphitization of biomass materials, therefore, represents an opportunity to upscale waste into functional nanomaterials. The pore structure depends on the morphology of the carbon template, resulting in great interest in the controlled production of biomass foams and gels. However, the graphitization process and resulting carbon morphology depends on the graphitization mechanism around different metal particles. By undertaking meta-analysis of different graphitization experiments, trends can be identified and compared with a new thermodynamic model to distinguish different behaviors from metal nanoparticles acting as graphitization catalysts.

## Results and Discussion

### Carbon Morphology

As discussed previously the model outlined by Oya and Marsh attribute different growth mechanisms to the different metal particles produced by the reduction process. G-effect graphitization from large, crystalline metal particles results in more graphitic carbon that can be distinguished by a sharp peak in XRD patterns with a smaller interlayer spacing (Figure S9). Smaller particles were linked to more turbostratic carbon, with a broader XRD peak at greater interlayer spacing. However, the development of electron microscopy and the growth of research following the experimental isolation of graphene has revealed such turbostratic carbon growth from metal nanoparticles is more varied than this model suggests.

**Figure 1:**
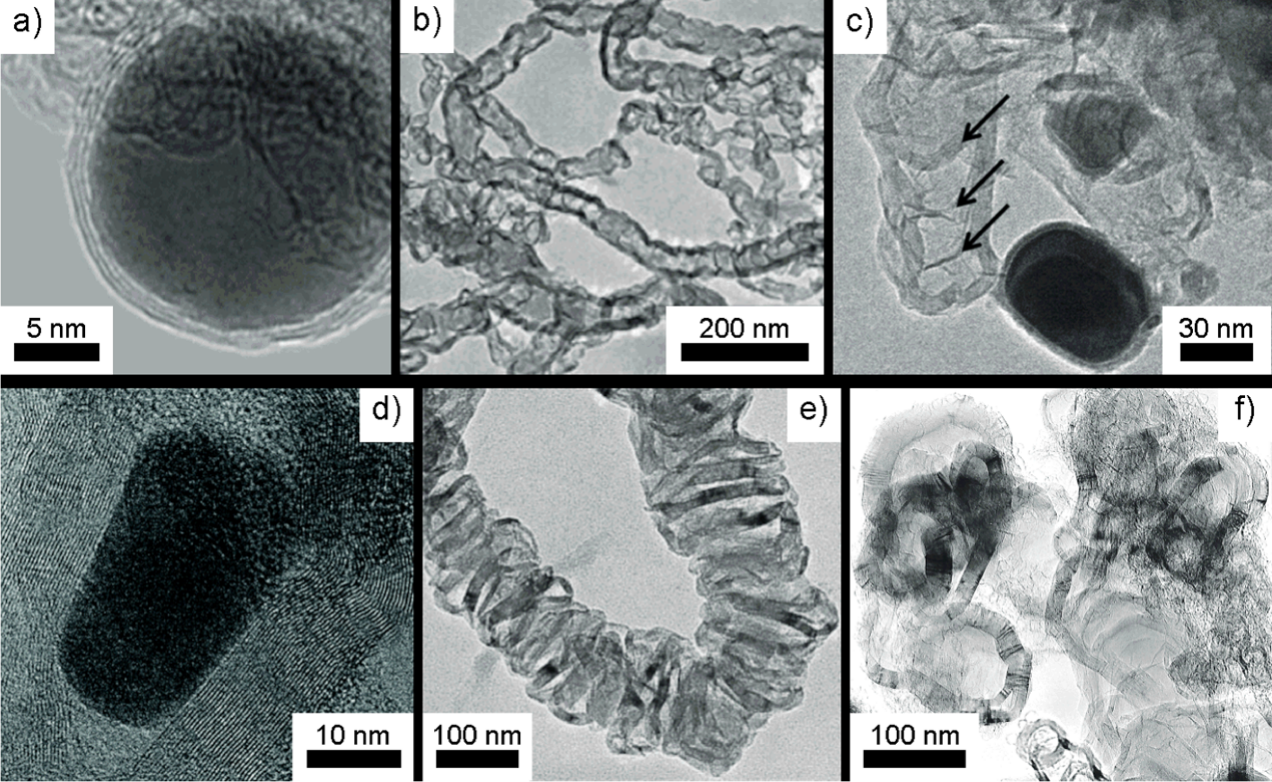
TEM images of different carbon morphologies. (a,d) metal particles inside carbon onions. (b) rough carbon tube, (c) growth process responsible for “bamboo” like tubes, and (e,f) carbon ribbons. 1a reprinted by permission from Springer, Journal of Nanoparticle Research, Catalytic graphitization of kraft lignin to graphene-based structures with four different transitional, Qiangu Yan et al., 2018. Copyright 2018. 1b reprinted from Carbon, 46, M. Sevilla, C. Sanchís, T. Valdés-Solís, E. Morallón, A.B. Fuertes, Direct synthesis of graphitic carbon nanostructures from saccharides and their use as electrocatalytic supports, 931–939, Copyright 2008 with permission from Elsevier. 1c reproduced from ref (13) with permission from the Royal Society of Chemistry. 1d reproduced from ref (40) with permission from the Royal Society of Chemistry. 1e reprinted from Materials Chemistry and Physics, 113, Marta Sevilla, Antonio B. Fuertes, Easy synthesis of graphitic carbon nanocoils from saccharides, 208–214, Copyright 2009 with permission from Elsevier. 1f adapted with permission from F. J. Maldonado-Hódar et al., *Langmuir***2000,***16,* 4367. Copyright 2001 American Chemical Society.

To describe the morphology of the carbon produced many descriptors are used: “nanocoils”,^6,12,16,17^ “nanoribbons”,^5,12,32,34,51,52^ “nanopipes”,^16^ “bamboo-like tubes”,^10,13^ “nanotubes”,^13,52,53^ “carbon-onions”,^10,34,54,55^ and “nanocapsules”.^16,34^ Many terms are used interchangeably by different researchers for the same morphology but there is also significant variation between some of these terms. “Carbon-onions” are small particles containing concentric layers of graphitic carbon, examples are shown in Figure 1a,d.^55^ “Nano-capsules” are also used to describe the same structure, although often for larger particles with more carbon layers.

“Nano-pipes”, “nanotubes”, and “bamboo-like tubes” all have a very similar morphology of tubular graphitic carbon pushed through the amorphous carbon matrix, as shown in Figure 1b and c. The significant difference prompting the distinction of “bamboo-like” tubes is the growth of carbon layers across the internal diameter of the tube, as seen in Figure 1c.^37^ “Nanoribbons” and “nanocoils” are thicker graphitic structures lacking a tubular structure, as shown in Figure 1e,f; the limitations of the 2D projection offered by TEM images suggest these could be the same structures that are sometimes observed bundled up with a pattern that can appear coiled. Despite the number of different terms, a clear distinction can be made between two fundamentally different behaviors. There is static growth producing uniform symmetrical carbon around a central particle, and carbon produced from a mobile particle with asymmetric growth in one direction as the metal particle has migrated through a structure.

**Figure 2:**
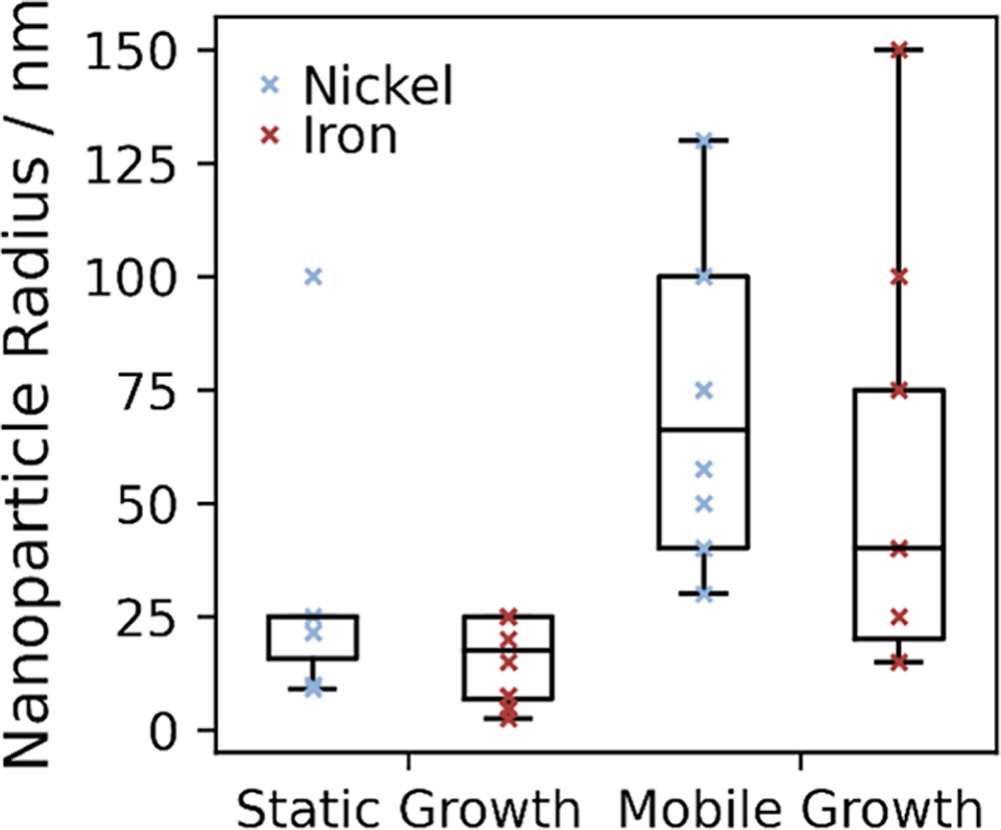
Box plot of maximum sizes of Fe and Ni metal particles observed to grow different carbon morphologies; morphologies assigned by subjective consideration of microscopy images. Values taken from refs (5)–^8^^10^,–^17^^20^^21^^32^,–^34^^40^^51^,–^52^^53^^56^,–^57^^58^^59^^60^^61^.

To investigate further, meta-analysis of graphitization literature was undertaken. The lack of reliable standard terms and methodologies between material preparations means no clear set of metrics can be employed; so to investigate the role of particle size, TEM images were used. From these, the carbon was subjectively described as one of the two fundamentally different growth mechanisms above, static or mobile growth. Box plots, as shown in Figure 2, of the maximum particle radius observed in TEM images with the carbon morphology produced reveals a clear correlation between small particles (*r* < 25 nm) and immobile growth, while larger particles usually resulted in a trail of graphitic carbon from a mobile metal particle. Fe particles 15–25 nm in radius may show a greater tolerance, with both carbon morphologies observed to grow in the few reports sufficiently characterized.

**Figure 3:**
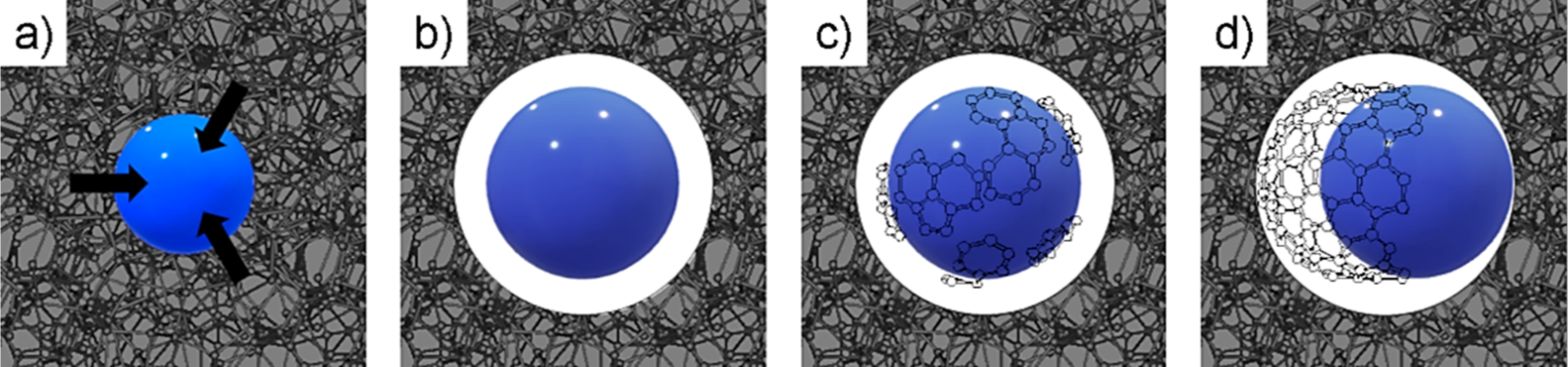
Schematic of the initial growth process. (a) Metal particle trapped within the carbon matrix will dissolve carbon around itself to form a solid solution. (b) As the density of the resulting quasi-carbide phase is greater, an empty space is created around the nanoparticle. (c) Carbon atoms freely migrate on the surface of the metal, forming into stable graphenic islands. (d) Complete hemisphere of graphenic carbon must leave the particle surface if stable nanotube growth can occur.

Reconciling these observations, the size of the metal particles and their high carbon solubility are likely to be crucial factors in controlling the carbon produced. When metal salts with low thermal stability are used in a dense carbon matrix, these particles can be become trapped. After carbothermal reduction, the hot metal will dissolve the surrounding carbon, producing a quasi-molten mixture of metal and carbon with a greater density than the original separate phases. This creates a vacant space around the metal particle from the carbon dissolution into the metal particle, as illustrated in Figure 3. Alternatively, in open pores, these metal particles may begin migrating along the internal surfaces. Carbon atoms on the surface of the metal particle are free to migrate and form temporary structures, which form islands of stable, graphenic carbon as modeled from carbon nanotube growth.^62−64^

The key deviation in the growth process is, therefore, the ability of some particles to become mobile and graphitize carbon as they migrate; this requires graphitic carbon, stabilized on the metal surface, to leave and permit new graphitization on the vacated surface site. Considering multiwall carbon nanotube growth as a model, one can imagine a hemisphere of graphitic carbon grown around the metal particle; if this hemisphere remains in place further growth will encapsulate the particle resulting in a carbon onion. For nanotube growth to take place, this hemisphere must leave the metal surface so the particle can graphitize as it migrates through the structure, forming nanotube walls as it does so, as illustrated in Figure 3d. However, the carbon on the metal surface has a favorable interaction with the metal that must be overcome to remove the nucleated graphitic carbon and allow the particle to transition to mobile growth.

### Energy Change of Graphitization Process

The initial removal of surface carbon creates an energy cost to transition to mobile growth. As graphitization is a thermodynamically favorable process, this energy cost can be overcome by further graphitization of the carbon matrix, for example, extending a longer nanotube. However, as hypothesized above, small nanoparticles within the carbon matrix only have a limited free volume around themselves in which to grow graphitic carbon. We suggest that static and mobile growth modes are determined by the balance between the energy cost of removing the carbon from the metal surface and the favorable free energy change of initial graphitization within the volume available around the metal particle.

We, therefore, considered a thermodynamic description of the system of multiwalled nanotube growth from an ideal spherical metal particle. The energy cost of removing the hemisphere can be approximated by the number of carbon atoms present using the area density of carbon in a graphitic lattice (), the radius of metal particle (*r*_m_), typical metal–carbon interaction distance (*d*_m_), and the interaction energy per carbon between the graphitic carbon and the metal (μ_m_).^65,66^ This simple model neglects the entropy change expected when removing the carbon from the metal surface for computational feasibility. The free energy change of multiwalled nanotube growth can then be calculated in terms of the outer radius (*r*_o_) and length of nanotube (*l*); using the free energy change for converting from amorphous carbon to turbostratic graphite and accounting for the surface energy created by the new nanotube and the strain of bending graphene flakes into the nanotube.

The free energy change per carbon atom transformed from amorphous into turbostratic carbon nanotubes was reported to be μ_G_ = −0.065 eV.^67^ The favorable energy change is reduced by the strain and surface energies of the newly created nanotubes, where the surface energy of nanotubes has been previously reported, σ = 0.4806 eV/nm^2^.^68,69^ Assuming perpendicular nanotube growth,^70^ the outer radius of the nanotube (*r*_o_) is set equal to the radius of metal nanoparticle (*r*_m_) and the inner radius (*r*_i_) is limited by the conservation of mass. The strain of the nanotubes can be calculated from a modified expression from Diaz et al. and taking α = 2.14 eV/nm^2^.^71,72^ This expression follows the derivation of Tibbetts by taking sheets of graphite and bending them into tubes;^73^ in this case, we are interested in a small number of layers in the nanotube so an exact summation is required, where the spacing between tubes is assumed to be comparable to graphite’s interlayer spacing (*a* = 3.35 Å).

Thus, considering the free energy change of graphitization, accounting for the strain and surface tension of the nanotubes created and the energy of removing a hemisphere of graphitic carbon, we obtain an approximate expression for the free energy change of the growth process in terms of particle size and nanotube length (eq 1), for a full derivation see the Supporting Information.
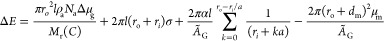
1

This can be solved to obtain the length of nanotube growth for a given particle radius at which the free energy change is zero, effectively identifying the minimum nanotube length required to overcome the energy barrier imposed by removing the carbon from the metal surface. This nanotube length is then compared with the free space available (*t*) around the particle which can be derived from the solubility of carbon in the metal (*x*) and the relative atomic volumes () of the carbon, metal, and the metal carbide formed (Equation 2).
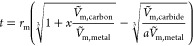
2

**Figure 4:**
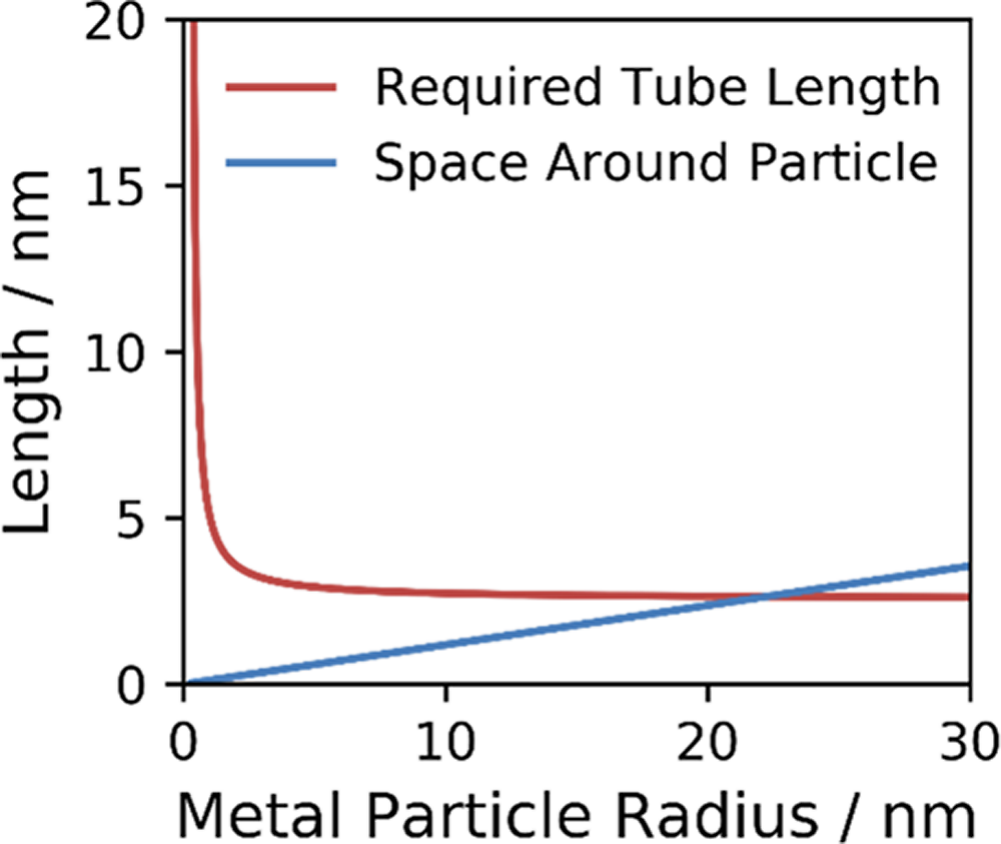
Model of nanotube growth from nickel nanoparticles. The minimum nanotube length required to overcome the energy barrier and sustain mobile growth shown in red, with the thickness of the empty shell around the nanoparticle shown in blue. The intersection at 22.2 nm is the minimum particle size capable of overcoming the energy barrier and sustaining mobile growth.

These terms are plotted in Figure 4, which shows a rapid decrease in nanotube length required as the particle radius increases, while the free space around the particle increases linearly. The intersection point is the minimum radius of nanoparticles with sufficient free volume around itself to form a nanotube long enough to overcome the energy cost of starting mobile growth. Particles smaller than this do not have enough space to graphitize sufficient carbon to overcome the energy cost of removing carbon from the metal surface, and so complete encapsulation is more favorable for these small particles. For Ni, this minimum particle size for mobile growth is calculated to be 22.2 nm and for Co 21.2 nm, plots for all metals shown in Figures S4–S6. Iron’s weaker carbon-metal interaction and lower density make the calculated minimal particle smaller, with an intersection at 15.9 nm, which supports the general observation that Fe particles tend to favor nanotube growth over graphene flakes.

**Figure 5:**
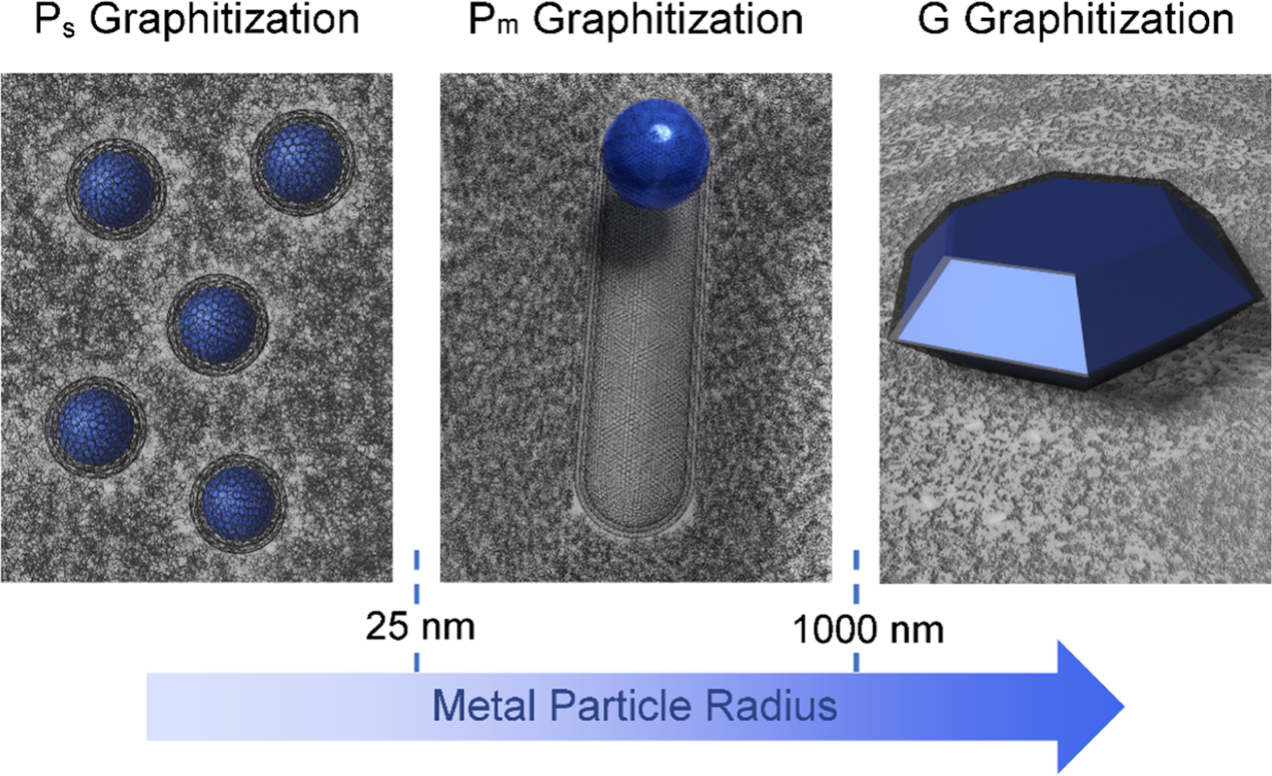
Illustration of the three main catalytic carbonization processes caused by the increasing size of metal particles. Nanoparticles smaller than 25 nm in radius remain static, growing carbon onions around themselves (*P*_s_-effect). Intermediate particles become mobile, producing tubes and ribbons pushed through the carbon matrix (*P*_m_-effect) while large micron scale metal particles precipitate crystalline graphite on their surfaces (G-effect).

Despite the simplicity of this model, the intersection point for these metals is in good agreement with the meta-analysis undertaken, which suggested particles less than 25 nm favor static growth, forming carbon onions and shells. These growth processes are in line with the observed morphologies described in Oya and Marsh’s original model; however, their *T*_s_-effect graphitization encompasses both nano-onion growth and tube and ribbon growth. It, therefore, seems appropriate to amend the original growth model to include the dynamics possible from nanoparticles. We propose the following description of different graphitization mechanisms, as illustrated in Figure 5.

A-effect graphitization, the selective reaction and removal of defective regions, is distinct from the catalytic graphitization discussed here and remains a valid approach. Indeed, a recent report using Zn appeared to be well described by this mechanism in contrast to claims of surface templating of graphitic carbon.^74^ G-effect describes the production of graphite crystals as a result of very large metal particles of the order of microns in size that are unable to melt and migrate through the carbon structure. These form solid solutions of carbon at high temperatures and produce highly crystalline graphite on subsequent cooling. *P*_s_, or static particle growth describes the mechanism of particles trapped within the carbon and too small to generate sufficient free space to sustain nanotubes or ribbons, instead these particles encase themselves in graphitic carbon shells.

*P*_m_ or mobile particle growth describes the process of larger particles that migrate through the carbon leaving a trail of graphitic carbon behind them. When trapped within a carbon matrix, these particles are capable of dissolving sufficient amorphous carbon to support mobile growth. This space is initially required to graphitize enough carbon to compensate for the energy cost of breaking the favorable carbon–metal interaction. Any particles on the surface of open pores should also be described by mobile growth because the free space required to begin migrating is always available. Nevertheless, our meta-analysis found very few examples of small, mobile particles. The open space within the pore may explain this trend; however, because any space large enough to support mobile graphitization is also likely to support the annealing of metal particles.

## Conclusions

Direct observation of graphitization remains challenging, however, by considering common findings from many in situ and ex situ observations, we present a thermodynamic model to explain the different growth behaviors observed from metal particles of different sizes. The overall placement and growth of metal particles and subsequent graphitization and resulting structure is clearly a complex interplay between different, likely competing, factors. Our thermodynamic model to describe one such parameter can, therefore, be used as a framework for further study and development. By focusing on the particle size as a determining factor between mobile and static growth, we have derived an expression to quantify this energy barrier and propose an updated taxonomy of graphitization trends. G-effect graphitization occurs from larger, micron scale crystalline metals that dissolve a lot of carbon within the particle and precipitate crystalline graphite. *P*_m_, or mobile particle growth, is observed from nanoparticles larger than 25 nm that likely anneal within more open structures and have the freedom to migrate through a carbon material leaving a trail of graphitic carbon. Finally, *P*_s_, or static particles, are nanoparticles smaller than 25 nm trapped within the carbon matrix that produce graphene shells around themselves. These mechanisms are in close agreement with the meta-analysis undertaken and provide a description of different graphitization behaviors observed from metal particles during processes that can be used to upcycle carbon waste into functional materials.

## Supporting Information Available

Derivations of the models used, including consants, additional plots for all metals modeled and meta-analysis discussed and illustrative characterization of graphitic and turbostratic carbon (PDF)
Database of nanoparticle metrics (XLSX)

## Abbreviations

TEM: transmission electron microscopy
XRD: X-ray diffraction
