# Supplementary material for: Graphitization by Metal Particles
Source: ACS Omega. 2023 Jan 12;8(3):3278–85. doi: 10.1021/acsomega.2c06848 (PMC9878637; doi:10.1021/acsomega.2c06848)
Supplement: Supplementary file 1 — ao2c06848_si_001.pdf [file ao2c06848_si_001.pdf]

Graphitization by Metal Particles - Supporting Information

Stuart J Goldie[1†] and Karl S Coleman*[2]

1. Department of Chemistry, Durham University, South Road, Durham, DH1 3LE, UK
2. School of Physical Sciences, Liverpool University, Peach Street, Liverpool, L69 7ZE, UK; karl.coleman@liverpool.ac.uk

KEYWORDS Graphene Foam, Graphitization Mechanism, Hierarchical Foam, Carbonization

ABSTRACT: Graphitization of carbon offers a promising route to upcycle waste biomass and plastics into functional carbon nanomaterials for a range of applications including energy storage devices. One challenge to the more widespread utilization of this technology is controlling the carbon nanostructures formed. In this work we undertake a meta-analysis of graphitization catalyzed by transition metals; examining the available electron microscopy data of carbon nanostructures and finding a correlation between different nanostructures and metal particle size. By considering a thermodynamic description of the graphitization process on transition metal nanoparticles we show an energy barrier exists that distinguishes between different growth mechanisms. Particles smaller than ~25 nm in radius remain trapped within closed carbon structures while nanoparticles larger than this become mobile and produce nanotubes and ribbons. These predictions agree closely with experimentally observed trends and should provide a framework to better understand and tailor graphitization of waste materials into functional carbon nanostructures.

Contents

Derivation of Free Space Around Metallic Sphere S2

Derivation of Energy Change of Graphitization S5

Intersection Points of Different Metals S10

Comparison of Metals and Conditions S12

Example Analysis of Graphitic and Turbostratic Graphitization S13

References S13

† Present Address: Physical Chemistry of Nanomaterials, Kassel Universität, Heinrich-Plett-Str. 40, 34132, Germany;

1. Derivation of Free Space Around Metallic Sphere

Basic definition of molar volume ($\tilde{V}_m$) where V is volume, n the number of moles of material, M_r the molar mass of material and ρ density.

$$\tilde{V}_m \equiv \frac{V}{n} \equiv \frac{M_r}{\rho} \quad \text{(Eqn.1)}$$

Define carbon solubility as a ratio of dissolved carbon relative to metal.

$$x = \frac{n_{carbon}}{n_{metal}} \quad \text{(Eqn.2)}$$

The volume of carbon dissolved in metal can be stated as:

$$V_{carbon} = \tilde{V}_{m,carbon} n_{carbon} \quad \text{(Eqn.3)}$$

$$V_{carbon} = \tilde{V}_{m,carbon} x n_{metal} \quad \text{(Eqn.4)}$$

Starting from a sphere of pure metal, Figure S 1a, assume a symmetrical shell of carbon is dissolved from around the metal, Figure S 1b, leaving a total volume of radius r_t. The metal-carbon phase formed, denoted carbide, may increase in size relative to the metal sphere leaving a small shell of empty space of thickness t, shown in Figure S 1c.

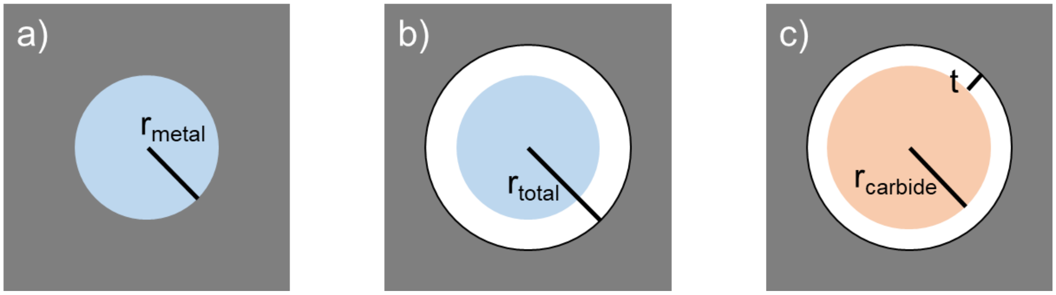

Figure S 1: Schematic of the different parameters used to model free space around metal particles embedded in carbon. a) Initial state of solid metal sphere (blue) in carbon (grey). b) Free space formed from carbon removed around metal particle with total sphere described by radius r_{total}. c) Metal-carbon phase increased in size relative to the initial metal particle; assuming the mixed metal-carbon has a greater density than the carbon dissolved resulting in a symmetrical shell of thickness, t, around the mixed

particle. This mixed phase is referred to herein as 'carbide' although this is not necessarily a known stable carbide phase.

The 'total volume' shown in Figure S1b can be calculated as a sum from the metal and carbon volumes, where the metal volume is a sphere and the carbon an outer shell.

$$V_{total} = V_{metal} + V_{carbon} \quad \text{(Eqn.5)}$$

Thus, the total radius of the metal particle and dissolved carbon can be written as:

$$r_{total}{}^{3} = \frac{3}{4\pi}\left(V_{metal} + V_{carbon}\right) \quad \text{(Eqn.6)}$$

Considering the two components, combining (Eqn.4) with the definition of metal molar volume gives an expression relating the volumes of the two; the pure metal sphere and the carbon shell.

$$\frac{V_{metal}}{\tilde{V}_{m,metal}} = \frac{V_{carbon}}{x\tilde{V}_{m,carbon}} \quad \text{(Eqn.7)}$$

Rearranging gives an expression for V_{carbon} in terms of metal particle radius.

$$V_{carbon} = \frac{4}{3}\pi x \frac{\tilde{V}_{m,carbon}}{\tilde{V}_{m,metal}} r_{metal}{}^{3} \quad \text{(Eqn.8)}$$

Substituting (Eqn.8) into (Eqn.6) and writing V_{metal} in terms of spherical radius produces:

$$r_{total}{}^{3} = r_{metal}{}^{3}\left(1 + x\frac{\tilde{V}_{m,carbon}}{\tilde{V}_{m,metal}}\right) \quad \text{(Eqn.9)}$$

To consider the size of the metal-carbon (carbide) formed we can describe this process according to the chemical equation:

$$a\mathrm{M} + \mathrm{C} \rightarrow \mathrm{M}_{a}\mathrm{C}$$

Using the metal content and molar volume of carbide formed, the change in volume compared with the original metal sphere can be calculated.

$$V_{carbide} = \tilde{V}_{m,carbide} n_{carbide} \text{ and } n_{carbide} = \frac{1}{a} n_{metal} \quad \text{(Eqn.10)}$$

$$\frac{a \cdot V_{carbide}}{\tilde{V}_{m,carbide}} = \frac{V_{metal}}{\tilde{V}_{m,metal}} \quad \text{(Eqn.11)}$$

Once again rewriting in terms of radius

$$r_{carbide}{}^3 = r_{metal}{}^3 \frac{\tilde{V}_{m,carbide}}{a\tilde{V}_{m,metal}} \quad \text{(Eqn.12)}$$

This thickness of the empty shell around the carbide particle (Figure S 1c) is then found by the difference between the radius of the total system and the radius of the expanded carbide phase. The linear distance from the edge of the particle to the carbon matrix, within which a nanotube can grow is then $2t$ as illustrated in Figure S 2.

$$t = r_{total} - r_{carbide} \quad \text{(Eqn.13)}$$

$$t = r_{metal} \left(\sqrt[3]{1 + x \frac{\tilde{V}_{m,carbon}}{\tilde{V}_{m,metal}}} - \sqrt[3]{\frac{\tilde{V}_{m,carbide}}{a\tilde{V}_{m,metal}}} \right) \quad \text{(Eqn.14)}$$

The molar volumes are calculated from the densities and thermal expansion coefficients listed in

Table S 1.

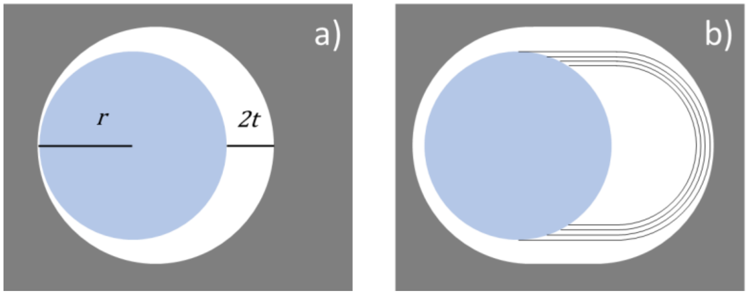

Figure S 2: Schematic of the empty shell, thickness t, around a particle facilitating nanotube growth. a) defines the particle radius and thickness of the empty shell around the particle whilst b) illustrates the growth process within the empty volume.

Table S 1: Density and thermal expansion coefficient values use for modelling the volume around the particles. The volumetric thermal expansion coefficients are taken as average values between 30°C and 1000 °C where possible; this neglects the actual function for simplicity since (Eqn.14) is insensitive to small changes in *ß*. Where no reference given, multiple sources compiled from Reaxys.

Compound	Density / $g\ nm^{-3}$	Volumetric Thermal Expansion Coefficient / K^{-1}
Carbon (amorphous)	1.5×10^{-21} [1]	4.5×10^{-6} [2]
Carbon (graphitic)	2.2×10^{-21} [1]	1.33×10^{-5} [3]
Nickel	8.9×10^{-21}	5.31×10^{-5} [4]
Nickel Carbide (Ni_3C)	8.08×10^{-21} [5]	*Ni value used*
Cobalt	8.9×10^{-21}	5.04×10^{-5}
Cobalt Carbide (Co_3C)	8.62×10^{-21} [6]	*Co value used*
Iron(γ-fcc)	7.60×10^{-21} [7]	*Iron ρ at 1000 °C*
Iron Carbide (Fe_3C)	7.7×10^{-21} [8]	4.1×10^{-5} [8]

2. Derivation of Energy Change of Graphitization

To describe the energy change of growing graphitic carbon nanotubes within the carbon matrix we consider four terms: the surface energy of the nanotube created, the strain energy of the nanotube, the energy of graphitization from converting amorphous carbon to graphitic, and the energy cost of removing the carbon hemi-sphere from the metal particle.

First the surface energy of the nanotubes created; where l is the length of nanotube, σ is the surface tension, and r the inner and outer radius of the tubes (Figure S 3). This term is trivial to derive simply multiplying the surface tension ($\sigma = 0.481\ eV/nm^2$) [1,9] with the inner and outer areas of the cylinder.

$$E_s = (\pi 2 r_0 l + \pi 2 r_i l)\sigma = 2\pi l(r_o + r_i)\sigma \qquad \text{(Eqn.15)}$$

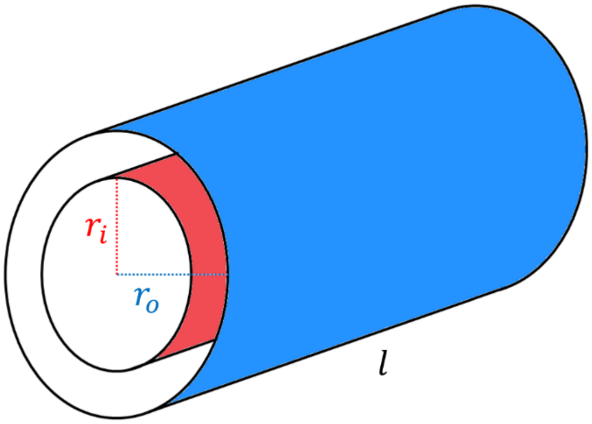

Figure S 3: Schematic of the inner and outer surface areas of nanotubes. Inner radius r_i, outer radius r_o and tube length l.

The outer radius of the growing nanotubes can be defined by the radius of the metal particle, assuming perpendicular growth from the edge of the particle. This assumption would be difficult to justify for CVD grown nanotubes which often show tangential growth of small SWCNTs; however, all TEM images reported of graphitization within a carbon matrix show a close match between the particle size and tube diameter.

The tube's inner diameter can then be defined from conservation of mass. As the particle migrates through the amorphous carbon a volume of amorphous carbon (V_a) is converted to graphitic carbon, which has a greater density. Subscripts a and g are used throughout to denote amorphous and graphitic carbon.

$$V_g \rho_g = m = V_a \rho_a \quad \text{(Eqn.16)}$$

$$l\pi({r_o}^2 - {r_i}^2) = l\pi {r_o}^2 \frac{\rho_a}{\rho_g} \quad \text{(Eqn.17)}$$

$${r_o}^2 \left(1 - \frac{\rho_a}{\rho_g}\right) = {r_i}^2 \quad \text{(Eqn.18)}$$

Assuming the outer radius and volume of carbon contained within the nanotube are both determined by the radius of the metal particle; the curvature becomes independent of the nanoparticle size, depending instead on the ratio of carbon density between the amorphous and graphitic carbon.

Another significant factor in the total energy change is the strain energy of rolling graphite into a tube. This can be calculated using the Young's modulus (E). Treating the tube wall as a solid block of graphite, the moment (M) required to bend the block through a moment of inertia (I) is given in terms of modulus (Y) and radius of curvature (R).

$$M = YI_y/R \qquad \text{(Eqn.19)}$$

For a block of thickness a and length L, where L will become the length of the carbon nanotube, the moment of inertia (I) can be written:

$$I_y = La^3/12 \qquad \text{(Eqn.20)}$$

The work (dw) required to bend such a block through an angle $d\theta$ is:

$$dw = \frac{1}{2}Md\theta \qquad \text{(Eqn.21)}$$

Integrating through a full circle and combining the above expressions results it the work required to bend such a block into a cylinder. This key equation was derived by Tibbetts.[10]

$$w = \frac{1}{2}M \times 2\pi = \pi Y \frac{La^3}{12R} \qquad \text{(Eqn.22)}$$

This is the energy of a single walled tube, the energy per carbon atom can then be found using the area density of carbon (Ω) and making the thickness equal to the layer spacing of graphite (a).[11] The distinction between R and r is very minor, where R refers the to the radius of curvature of a block whereas r refers to the specific radius of the carbon nanotube.

$$\frac{w}{n} = \frac{\pi Y L a^2}{12R} \div \frac{2\pi RL}{\Omega} = \frac{Ya^3\Omega}{24}\frac{1}{r^2} \qquad \text{(Eqn.23)}$$

This form can be used to derive the strain per carbon atom depending on the radius of the nanotube. Magnin et al. found this constant to be $\alpha = 2.14\ eV/\text{Å}^2$.[12,13] To clarify, the original paper erroneously states Ω as volume density rather than area density; however, a simple resolution of the units and derivation makes this clear.

$$E_c = \frac{Ya^3\Omega}{24}\frac{1}{r^2} = \frac{\alpha}{r^2} \quad \text{(Eqn.24)}$$

This expression gives the energy per atom for a single walled nanotube. To find the total strain, we must calculate the number of carbon atoms in each nanotube using the total nanotube area and area per carbon atom, $\tilde{A}_G = \frac{1}{\Omega}$. This expression is then summed over all the layers expected for the multiwalled nanotube created, where a is the graphite interlayer spacing.

$$E_c = \frac{2\pi\alpha l}{\tilde{A}_G}\sum_{k=0}^{\frac{r_o - r_i}{a}} \frac{1}{(r_i + ka)} \quad \text{(Eqn.25)}$$

The third consideration for the total free energy change of the metal particle graphitization is the energy of converting amorphous carbon to graphitic carbon. The free energy change per carbon atom for this transformation was previously reported to be $\Delta\mu_g = 0.065\ eV$.[14] The volume of amorphous carbon converted is a cylinder described above; from this the number of carbon atoms can be calculated from the molar mass ($M_r(C)$) and density (ρ_a) of amorphous carbon.

$$\Delta E_g = \Delta\mu_g \pi {r_o}^2 l \frac{\rho_a N_a}{M_r(C)} \quad \text{(Eqn.26)}$$

Finally, we must consider the energy cost of removing the hemi-sphere of carbon bound to the metal particle, required for the nanotube to start growing outwards. The binding energy per carbon atom (μ_m) was taken from DFT calculations and are shown in Table S2.

Assuming the binding energy is only significant for the first layer, the number of carbon atoms can be calculated from the area of the hemi-sphere and the area density of carbon ($\tilde{A}_G$). Here the radius of the hemisphere is taken to be the outer radius of the metal particle (r_o) plus the equilibrium distance between the metal surface and carbon layer (d_m); values taken from literature and listed in Table S2.

Table S 2: Data table of binding energy and equilibrium interaction distance for graphene on different transition metal surfaces. The averaged values are used for spherical particles. Data for Ni and Co taken from Ref. [15], Fe taken from Ref. [16]

Metal Surface	Binding Energy / eV per carbon	Metal Carbon Distance / nm
Ni(111)	0.135	0.202
Ni (110)	0.178	0.200
Ni(100)	0.175	0.216
Ni Average	0.163	0.206
Co(111)	0.259	0.198
Co(110)	0.221	0.203
Co(100)	0.162	0.221
Co Average	0.214	0.207
Fe(110)	0.149	0.211

$$\Delta E_{sorption} = \frac{2\pi(r_o + d_m)^2 \mu_m}{\tilde{A}_G} \quad \text{(Eqn.27)}$$

This term calculates the enthalpy of removing the carbon from the metal surface, however neglects the entropic change from this process. An accurate description of the relative populations of all the vibrational micro-states of the carbon hemi-sphere, with and without the metal particles surface potential, is beyond the scope of this work. This omission is a limitation of the current model; however, this entropic contribution is likely to be small.

Combining these four contributions, equations: 15, 25, 26 and 27, produces an approximate free energy change of the system on graphitization in terms of the nanoparticle radius and tube length.

$$\Delta E = \frac{\pi {r_o}^2 l \rho_a N_a \Delta\mu_g}{M_r(C)} + 2\pi l(r_o + r_i)\sigma + \frac{2\pi\alpha l}{\tilde{A}_G} \sum_{k=0}^{\frac{r_o - r_i}{a}} \frac{1}{(r_i + ka)} - \frac{2\pi(r_o + d_m)^2 \mu_m}{\tilde{A}_G} \quad \text{(Eqn.28)}$$

We are interested in the minimal tube length required for growth to be favourable; so, setting $\Delta E = 0$ and re-writing in terms of l gives an expression for the minimal tube length required in terms of nanoparticle radius.

$$l = \frac{2(r_o + d_{cm})^2 \mu_m \frac{1}{\tilde{A}_G}}{2\sigma(r_o + r_i) + \frac{2\alpha}{\tilde{A}_G} \sum_{k=0}^{\frac{r_o - r_i}{a}} \frac{1}{(r_i + ka)} + \frac{r_o \rho_a N_a \Delta\mu_g}{M_r(C)}} \qquad \text{(Eqn.29)}$$

3. Intersection Points of Different Metals

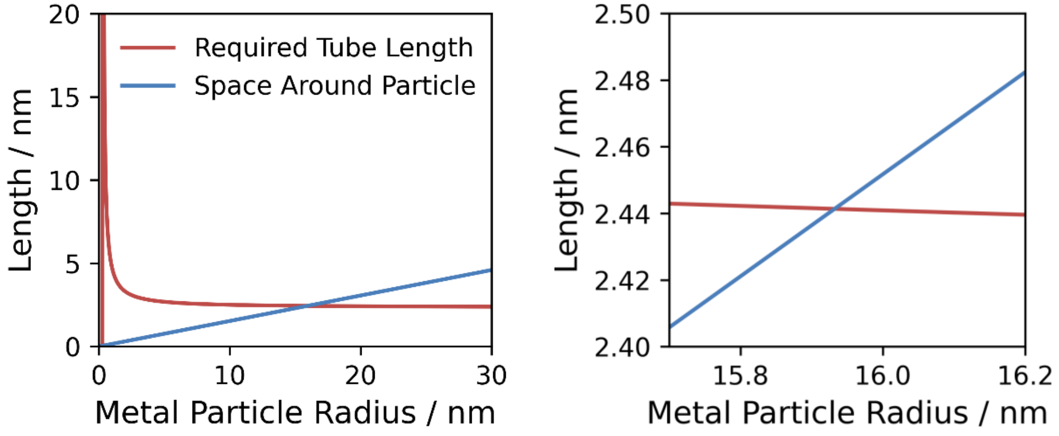

Figure S 4: Plot showing the intersection of the free volume and nanotube length required for mobile growth from iron nanoparticles; particles larger than 15.9 nm in radius are required for mobile growth.

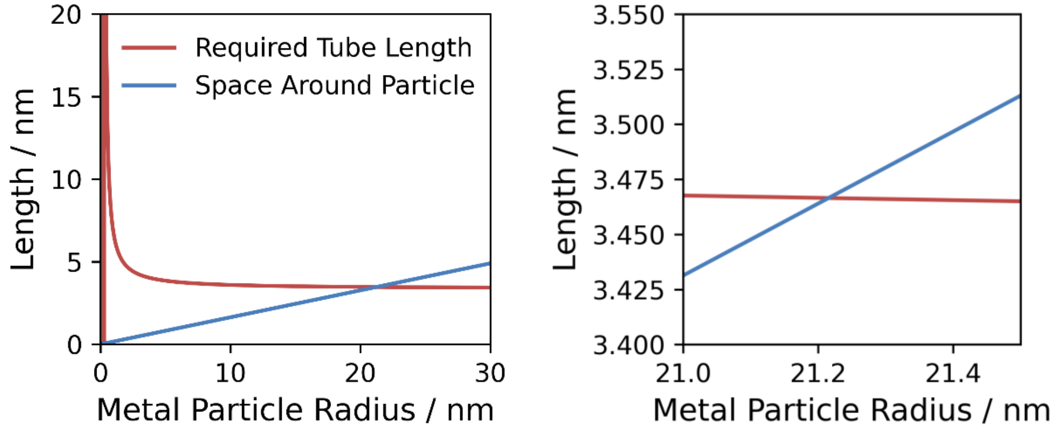

Figure S 5: Plot showing the intersection of the free volume and nanotube length required for mobile growth from cobalt nanoparticles; particles larger than 21.2 nm in radius are required for mobile growth.

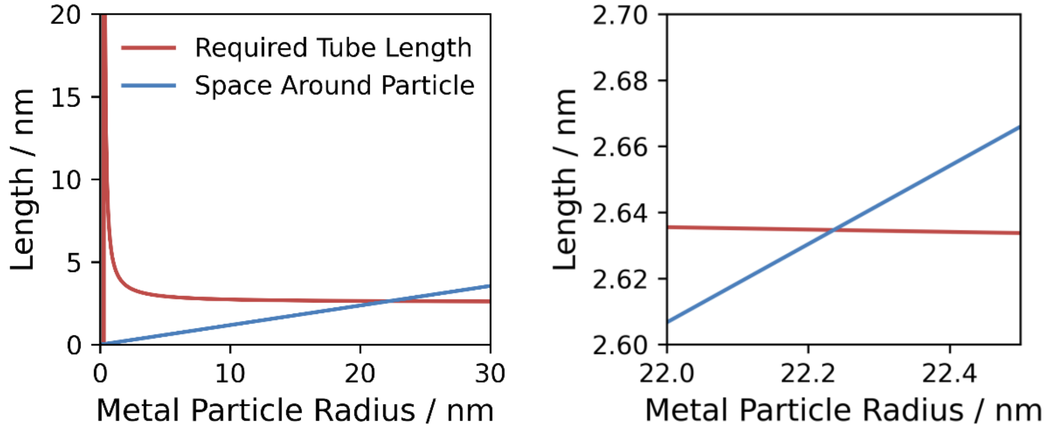

Figure S 6: Plot showing the intersection of the free volume and nanotube length required for mobile growth from nickel nanoparticles; particles larger than 22.2 nm in radius are required for mobile growth.

4. Comparison of Metals and Conditions

Considering different metals it is curious to note nickel produced larger particles (Figure S 7). Cobalt broadly followed the same trends as nickel, however the sample size was too small to reliably use. Iron particles were generally smaller in the literature reviewed for this work, with an upper quartile of 40 nm compared with almost double from nickel particles.

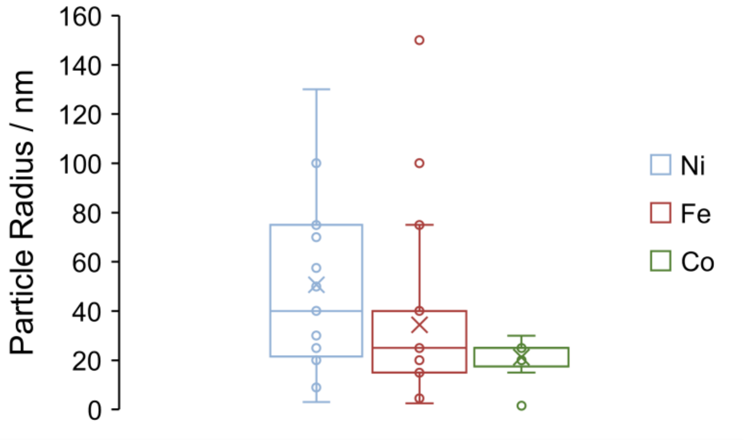

Figure S 7: Box plot of metal particle radius from different metals, particles were formed under a variety of different reactions conditions.

Examining the different carbon morphologies reported relative to the metal used as a catalyst we found nickel produced a greater proportion of nanoribbons and nanotubes from mobile growth (Figure S 8), 72% compared to just 48% of the samples from iron. However, considering the iron particles were generally smaller in the literature analysed for this report this remains consistent with the thermodynamics of the growth on different metals.

It is impossible from these observations alone to determine whether the particle size or metal is causing this trend; however, studies using different salts have and the thermal stability of the salt to be a key parameter in controlling the size and distribution of particles produced. More thermally stable salts form larger metal particles and crystalline graphite on their surface compared with less thermally stable salts forming nanoparticles.[17–19] Therefore, we conclude the particle size is determining the morphology of carbon produced.

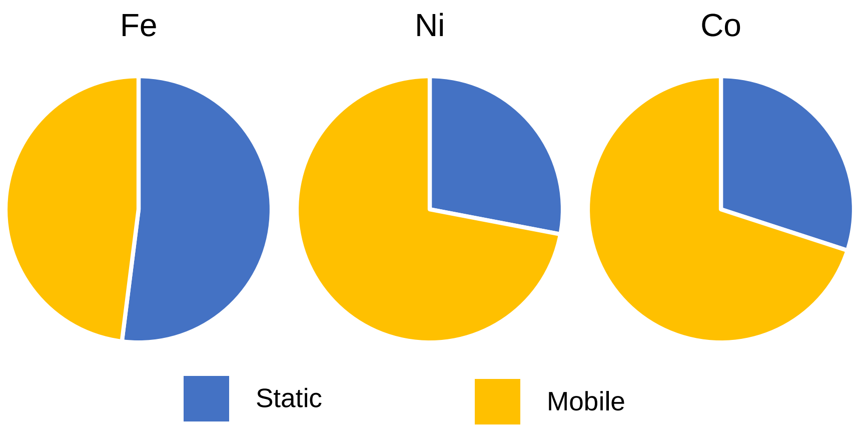

Figure S 8: Pie charts of the distribution of carbon morphologies observed from different metal catalysts.

5. Example Analysis of Graphitic and Turbostratic Graphitization

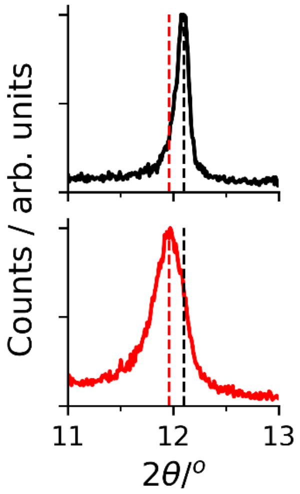

Figure S 9: Example carbon (002) XRD reflections from graphitic (black) and turbostratic (red) graphitization. Recorded with Mo Kα x-rays (λ = 0.7093 Å) from iron and cobalt catalysed foams following the method from ref [19].

6. References

(1) Ci, L.; Wei, B.; Xu, C.; Liang, J.; Wu, D.; Xie, S.; Zhou, W.; Li, Y.; Liu, Z.; Tang, D. Crystallization Behavior of the Amorphous Carbon Nanotubes Prepared by the CVD Method. *Journal of Crystal Growth* **2001**, *233* (4), 823–828. https://doi.org/10.1016/S0022-0248(01)01606-2.

(2) Champi, A.; Lacerda, R. G.; Viana, G. A.; Marques, F. C. Thermal Expansion Dependence on the Sp2 Concentration of Amorphous Carbon and Carbon Nitride. *Journal of Non-*

Crystalline Solids **2004**, *338–340*, 499–502. https://doi.org/https://doi.org/10.1016/j.jnoncrysol.2004.03.028.
(3) Zorzi, J. E.; Perottoni, C. A. Thermal Expansion of Graphite Revisited. *Computational Materials Science* **2021**, *199*, 110719. https://doi.org/https://doi.org/10.1016/j.commatsci.2021.110719.
(4) W, H. N. Jr.; Sisler, H. H. Determination of Residual Stresses in Titanium Carbide-Base Cermets by High-Temperature X-Ray Diffraction. *Journal of the American Ceramic Society* **1958**, *41* (3), 93–103. https://doi.org/https://doi.org/10.1111/j.1151-2916.1958.tb15449.x.
(5) Nagakura, S. Study of Metallic Carbides by Electron Diffraction Part II. Crystal Structure Analysis of Nickel Carbide. *Journal of the Physical Society of Japan* **1958**, *13* (9), 1005–1014. https://doi.org/10.1143/JPSJ.13.1005.
(6) Nagakura, S. Study of Metallic Carbides by Electron Diffraction Part IV. Cobalt Carbides. *Journal of the Physical Society of Japan* **1961**, *16* (6), 1213–1219. https://doi.org/10.1143/JPSJ.16.1213.
(7) Straumanis, M. E.; Kim, D. C. Lattice Constants, Thermal Expansion Coefficients, Densities and Perfection of Structure of Pure Iron and of Iron Loaded with Hydrogen. *International Journal of Materials Research* **1969**, *60* (4), 272–277. https://doi.org/doi:10.1515/ijmr-1969-600404.
(8) Wood, I. G.; Vočadlo, L.; Knight, K. S.; Dobson, D. P.; Marshall, W. G.; Price, G. D.; Brodholt, J. Thermal Expansion and Crystal Structure of Cementite, Fe3C, between 4 and 600K Determined by Time-of-Flight Neutron Powder Diffraction. *Journal of Applied Crystallography* **2004**, *37* (1), 82–90. https://doi.org/10.1107/S0021889803024695.
(9) Abrahamson, J. The Surface Energies of Graphite. *Carbon N Y* **1973**, *11* (4), 337–362. https://doi.org/https://doi.org/10.1016/0008-6223(73)90075-4.
(10) Tibbetts, G. G. Why Are Carbon Filaments Tubular? *Journal of Crystal Growth* **1984**, *66* (3), 632–638. https://doi.org/https://doi.org/10.1016/0022-0248(84)90163-5.
(11) Robertson, D. H.; Brenner, D. W.; Mintmire, J. W. Energetics of Nanoscale Graphitic Tubules. *Phys. Rev. B* **1992**, *45* (21), 12592–12595. https://doi.org/10.1103/PhysRevB.45.12592.
(12) Gülseren, O.; Yildirim, T.; Ciraci, S. Systematic Ab Initio Study of Curvature Effects in Carbon Nanotubes. *Phys. Rev. B* **2002**, *65* (15), 153405. https://doi.org/10.1103/PhysRevB.65.153405.
(13) Magnin, Y.; Amara, H.; Ducastelle, F.; Loiseau, A.; Bichara, C. Entropy-Driven Stability of Chiral Single-Walled Carbon Nanotubes. *Science (1979)* **2018**, *362* (6411), 212–215. https://doi.org/10.1126/science.aat6228.
(14) Kelly, B. T. *Physics of Graphite*; Applied Science London, 1981.
(15) Zhang, X.; Wang, S. First-Principles Investigation of the Microscopic Mechanism of the Physical and Chemical Mixed Adsorption of Graphene on Metal Surfaces. *RSC Adv.* **2019**, *9* (56), 32712–32720. https://doi.org/10.1039/C9RA07111C.
(16) Restuccia, P.; Righi, M. C. Tribochemistry of Graphene on Iron and Its Possible Role in Lubrication of Steel. *Carbon N Y* **2016**, *106*, 118–124. https://doi.org/https://doi.org/10.1016/j.carbon.2016.05.025.
(17) Hoekstra, J.; Beale, A. M.; Soulimani, F.; Versluijs-Helder, M.; van de Kleut, D.; Koelewijn, J. M.; Geus, J. W.; Jenneskens, L. W. The Effect of Iron Catalyzed Graphitization on the Textural Properties of Carbonized Cellulose: Magnetically Separable

Graphitic Carbon Bodies for Catalysis and Remediation. *Carbon N Y* **2016**, *107*, 248–260. https://doi.org/https://doi.org/10.1016/j.carbon.2016.05.065.

(18) Schnepp, Z.; Thomas, M.; Glatzel, S.; Schlichte, K.; Palkovits, R.; Giordano, C. One Pot Route to Sponge-like Fe3N Nanostructures. *J. Mater. Chem.* **2011**, *21* (44), 17760–17764. https://doi.org/10.1039/C1JM12842F.

(19) Goldie, S. J.; Jiang, S.; Coleman, K. S. Cobalt Nanoparticle Catalysed Graphitization and the Effect of Metal Precursor Decomposition Temperature. *Mater. Adv.* **2021**, *2* (10), 3353–3361. https://doi.org/10.1039/D1MA00125F.
